# MOFs-Based Nitric Oxide Therapy for Tendon Regeneration

**DOI:** 10.1007/s40820-020-00542-x

**Published:** 2020-11-11

**Authors:** Jun Chen, Dandan Sheng, Ting Ying, Haojun Zhao, Jian Zhang, Yunxia Li, He Xu, Shiyi Chen

**Affiliations:** 1 Department of Sports Medicine, Huashan Hospital, Fudan University, Shanghai, 200040 People’s Republic of China; 2 College of Chemistry and Materials Science, Shanghai Normal University, Shanghai, 200234 People’s Republic of China; 3 Department of Ultrasound, Jing’an District Center Hospital, Fudan University, Shanghai, 200040 People’s Republic of China

**Keywords:** Nitric oxide, Metal–organic frameworks, Tendon, Tissue regeneration, Angiogenesis

## Abstract

Tendon regeneration is still a great challenge due to its avascular structure and low self-renewal capability. The nitric oxide (NO) therapy emerges as a promising treatment for inducing the regeneration of injured tendon by angiogenesis. Here, in this study, a system that NO-loaded metal–organic frameworks (MOFs) encapsulated in polycaprolactone (PCL)/gelatin (Gel) aligned coaxial scaffolds (NMPGA) is designed and prepared for tendon repair. In this system, NO is able to be released in vitro at a slow and stable average speed of 1.67 nM h^−1^ as long as 15 d without a burst release stage in the initial 48 h. Furthermore, NMPGA can not only improve the tubular formation capability of endothelial cells in vitro but also obviously increase the blood perfusion near the injured tendon in vivo, leading to accelerating the maturity of collagen and recovery of biomechanical strength of the regenerated tendon tissue. As a NO-loaded MOFs therapeutic system, NMPGA can promote tendon regeneration in a shorter healing period with better biomechanical properties in comparison with control group by angiogenesis. Therefore, this study not only provides a promising scaffold for tendon regeneration, but also paves a new way to develop a NO-based therapy for biomedical application in the future.

**Electronic supplementary material:**

The online version of this article (10.1007/s40820-020-00542-x) contains supplementary material, which is available to authorized users.

## Highlights

- A system that NO-loaded metal–organic frameworks encapsulated in PCL/Gel aligned coaxial scaffold is successfully constructed.
- The system enables to release NO slowly (1.67 nM h^−1^) and stably in a long period (15 d) without a burst release in the initial 48 h.
- The scaffold can promote the regeneration of the injured tendon with maturer collagen fibers and better mechanical properties by angiogenesis.

**Electronic supplementary material:**

The online version of this article (10.1007/s40820-020-00542-x) contains supplementary material, which is available to authorized users.

## Graphical abstract

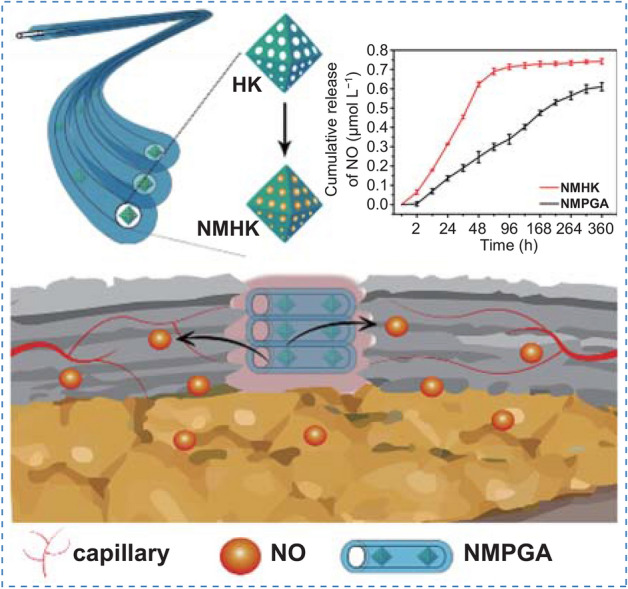

## Introduction

Tendons are the core component of the locomotor system, where injuries will lead to partial or complete loss of motor function [1]. Annually, there are over 15 million incidents of tendon injuries worldwide, even worse to disability [2]. Due to the fact that tendons are hypovascular tissue, it is very difficult for tendon regeneration because of the disruption of blood supply after tendon injury [3–5]. Therefore, the recovery of blood supply is very important during the repair period of injured tendons. Nowadays, the angiogenesis has been considered as one of the best strategies for recovering blood supply, because it could significantly increase the blood supply and rapidly improve the blood microcirculation around the region of injured tendon [6, 7]. Furthermore, the functional outcomes of regenerated tendons such as mechanical properties have also been reported to be significantly improved by angiogenesis in comparison with that in the natural healing process, which enables to obviously reduce even avoid the risk of tendon injury again [8–10]. Hence, the development of angiogenesis is widely treated as a very critical step for tendon regeneration now.

Nitric oxide (NO), as a therapeutic agent, has unique biological features in angiogenesis and has a great potential in biomedicine [11]. However, as an active gas molecule, the biomedical application of NO is now mainly limited by the problems of storage, transportation, and release [12]. Recently, biomaterial-based scaffolds, including electrospun films, hydrogels, and metal nanoparticles, have been recruited in the NO delivery [13, 14]. However, these scaffolds are still suffering the drawbacks such as low payload and burst release of NO [15]. Therefore, to develop a scaffold with the continuous and stable release of NO is an urgent need.

Fortunately, the metal–organic framework (MOF) has been widely applied in the field of gas storage because of its high porosity, large specific surface area, and especially, the abundant active sites which can combine with a lot of gas molecules stably [16–18]. Moreover, MOFs are biodegradable in protein-containing solutions, and the metal ions in MOFs can be released and exhibited unique biological activity in the body [19]. Here, owing to the promotion of angiogenesis of copper ion [20], as a representative Cu-based MOFs with abundant Cu ions, HKUST-1 (HK) is recruited as a precursor to load NO in this study.

**Scheme 1:**
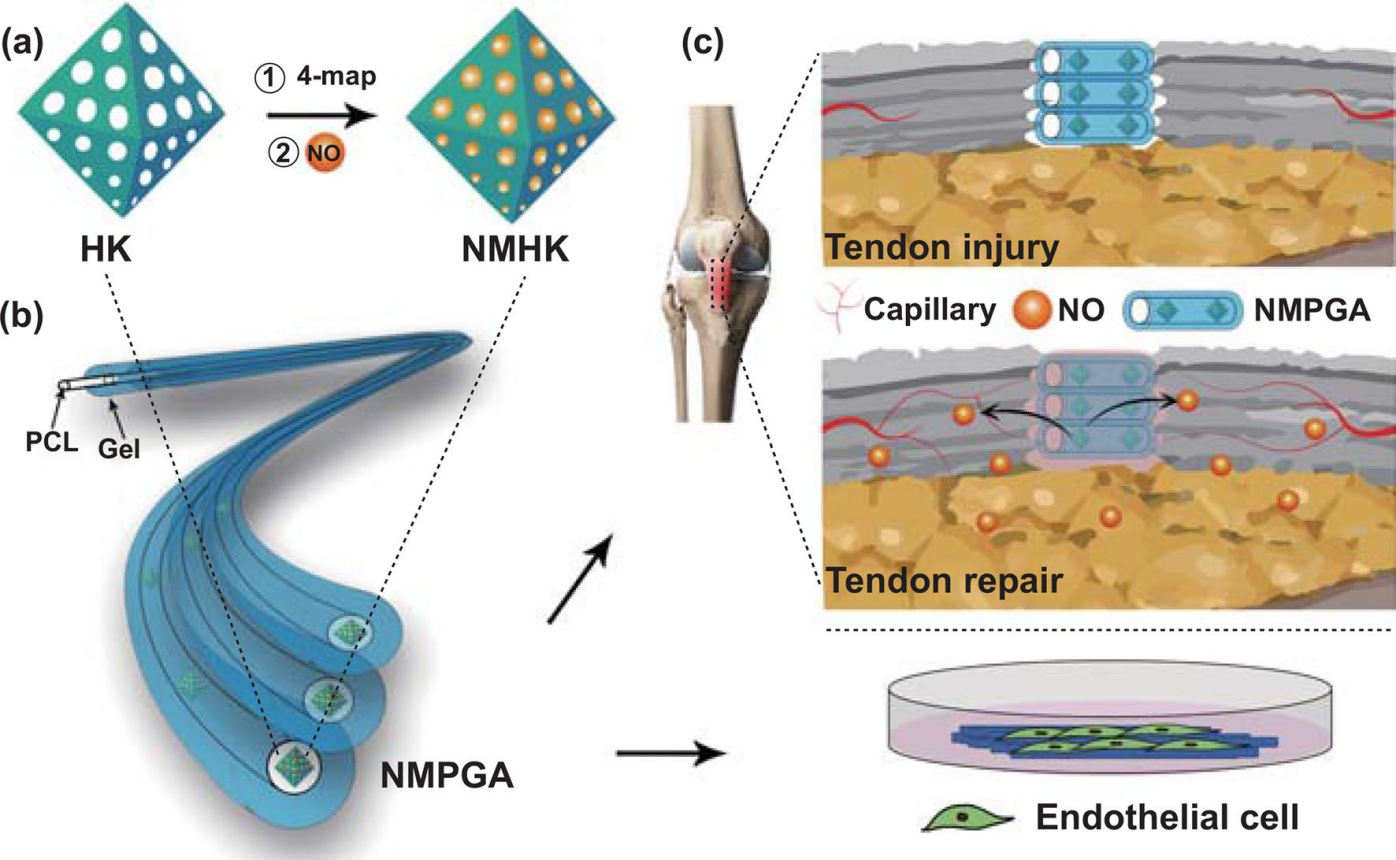
**a** Synthesis of NMHK.** b** Construction of NMPGA.** c** Application of NMPGA in vitro and in vivo

Herein, as shown in Scheme 1, a MOFs-based NO therapeutic system was designed and prepared in order to provide a local, stable, and sustained release of NO for tendon regeneration. To begin with, the payload of NO in HK was increased through the 4-(methylamino) pyridine (4-map) modification [21, 22]. Besides, our previous works confirmed that the polycaprolactone (PCL)/gelatin (Gel) aligned scaffolds can significantly enhance the biomechanical strength of the regenerated tendon by mimicking the organized native collagen fibers [23]. To further release NO slowly, aligned PCL/Gel scaffolds constructed with coaxial fibers were designed, and HK loaded with NO (NMHK) were encapsulated into the hydrophobic PCL cores of the coaxial fibers, which would separate the particles from the external water to prevent the undesired NO release. Moreover, angiogenic properties of the NO-loaded MOFs encapsulated in PCL/Gel aligned coaxial scaffolds (NMPGA) were comprehensively evaluated in vitro and in vivo*,* respectively.

## Experimental Section

### Materials

Poly (caprolactone) with average molecular weight of about 80 kDa, gelatin (gel strength ~ 250 Bloom), Hexafluoro-2-propanol (HFIP), 2,2,2-trifluoroethanol (TFE), Cu(NO_3_)_2_·3H_2_O (ACS, 98%), 1,3,5-Benzenetricarboxylic acid (H_3_BTC, C_9_H_6_O_6_, 98%), 4-(Methylamino) pyridine (4-map, C_6_H_8_N_2_, 99%) were all obtained directly from Aladdin Chemicals Reagent Co. (Shanghai, China). Ethanol (C_2_H_5_OH, 100%) was purchased from Richjoint Chemical Reagent Co. Ltd. (Shanghai, China). The Glutaraldehyde (C_5_H_8_O_2_, 25%) was purchased from Sinopharm Chemical Reagent Co. (Shanghai, China). Nitric Oxide (NO, 99.95%) was ordered directly from Zhonghao Guangming Chemical Gas Co. Ltd. (Shanghai, China). All the reagents and solvents were commercially available and used as received without any further purifying treatment.

### Synthesis of HK Nanoparticles

HKUST-1 nanoparticles were synthesized according to a modified method as previously reported [24]. In a typical process, Cu(NO_3_)_2_·3H_2_O (1.73 g, 9.06 × 10^–3^ M) and H_3_BTC (0.5 g, 2.38 × 10^–3^ M) were dissolved in a mixture of absolute ethanol and distilled water (9:1, v/v), respectively, naming as solution A and solution B. Then, both solutions A and B were cooled to − 60 ℃ by utilizing liquid nitrogen. After that, the solution A was poured into the solution B quickly, and the mixture solution was heated in a water bath (25 ℃). Finally, the light blue solid products were collected after centrifugation (10,000 rpm for 5 min), washed with ethanol for several times, and then dried at 60 ℃ overnight under vacuum to completely remove the residual solvent.

### Synthesis of NMHK Nanoparticles

Before NO loading, the secondary amino groups were introduced in HK. Firstly, the as-prepared HK (100 mg) was packed into a glass vial, and then transferred into a Teflon lined solvothermal autoclave (50 mL), which had been pre-filled with 4-map (35.4 mg, 0.327 × 10^–3^ M) [22]. After that, the autoclave was sealed carefully and heated at 140 ℃ for 12 h under autogenous pressure. Finally, the green solid products were obtained after being washed with ethanol for several times, centrifuged (10,000 rpm for 5 min), and dried at 60 ℃ overnight under vacuum to completely remove the residual solvent (Fig. S1). The 4-map modified HK nanoparticles were named as MHK. To load NO, the as-prepared MHK (50 mg) was heated at 120 ℃ for 10 h for thermal activation. After being cooled to room temperature, the MHK were transferred to a pressure apparatus. Firstly, the chamber of the pressure apparatus was pre-purged with argon for 30 min. Then, the samples were exposed to the dry NO under 2 atm for 1 h to load the NO in the MHK. After that, the chamber of the pressure apparatus was filled with argon for 30 min again to remove the residual NO. The green nanoparticles were collected and stored in the dry sealed condition to prevent the undesired leakage of NO.

### Fabrication of PGA, MPGA and NMPGA

The coaxial electrospinning technology was utilized to prepare the scaffolds. Firstly, as for the core layer, 0 wt% nanoparticle, 0.1 wt% MHK, 0.1 wt% NMHK were dispersed in a mixed solution of TFE and HFIP (2:3, v/v) and sonicated for several minutes, respectively. Then, 0.6 g PCL particles were added to the corresponding suspensions with continuous stirring to obtain a homogeneous electrospinning solution with a concentration of about 12% (w/v), respectively. Secondly, as for the shell layer, 0.45 g Gel was added in the mixed solution of TFE and HFIP (2:3, v/v) with continuous stirring to form the electrospinning solution with a concentration of about 9% (w/v). Then, a spinneret with concentric structure was chosen to carry out the electrospinning process with an electrospinning apparatus (TEADFS-103, Beijing, China). Correspondingly, the applied voltage was set at 15 kV. The solution feed rate of the core layer was 0.012 mL min^−1^, while the shell layer was 0.006 mL min^−1^. The distance between the tip of the spinneret and the drum collector was 18 cm, the speed of the collecting drum was 2000 r min^−1^, and the collecting time for all samples was fixed for 3 h. The whole experiments were conducted under room temperature, and the relative humidity was around 30 ~ 45%. The scaffolds with different kinds of additives (0 wt% nanoparticle, 0.1 wt% MHK, 0.1 wt% NMHK) were named as PGA, MPGA, and NMPGA, respectively. After the electrospinning process, all the as-prepared scaffolds were placed in a glass chamber, which was filled with glutaraldehyde saturated steam to cross-link for 3 h. After that, the scaffolds were immersed in the absolute ethanol overnight to eradicate the excess glutaraldehyde. Finally, all the scaffolds were dried in vacuum for 24 h to completely remove the residual solvent before further characterization.

### Morphology Observation

In order to observe the morphologies of the as-prepared MOFs (HK, MHK, NMHK) and scaffolds (PGA, MPGA, NMPGA), the field emission scanning electron microscopy (FE-SEM, Hitachi S-4800, Japan) was used. Before scanning, the surface of all the samples was carefully sprayed with gold according to the operation manual. Furthermore, transmission electron microscopy (TEM, Hitachi HT7800, Japan) was applied to observe the internal structure of the as-prepared MOFs and the scaffolds. The average diameter (*n* = 20) of the nanoparticles and nanofibers was measured by Image J software (NIH, USA).

### Physico-chemical Characterization

The XRD (D/max-2200, Rigaku, Japan) was used to exam the phases of the as-prepared MOFs (HK, MHK, NMHK) and scaffolds (PGA, MPGA, NMPGA) by using CuKα radiation (*ƛ* = 1.541874 Å, 40 kV, 100 mA) within the scanning range of 2*θ* = 5°–40° at a scanning rate of 2° min^−1^ and a step width of 0.02°. Additionally, the Fourier transform infrared spectra (FTIR, Frontier, USA) with the scanning wavenumber range of 4000 ~ 400 cm^−1^ was used to determine the functional groups of the samples at a resolution of 4 cm^−1^. Furthermore, the Raman spectroscopy (RM 1000, Renishaw, UK) was used to further verify the chemical composition of the as-prepared samples with the wavenumber ranging from 800 to 2000 cm^−1^. Moreover, in order to obtain the specific surface area and pore size of the HK and NMHK, the N_2_ adsorption–desorption measurements were taken by a surface area analyzer (ASAP 2020, Micromeritics Co., USA) at 77 K. The specific surface areas were calculated by the Brunauer–Emmett–Teller (BET) method according to the adsorption isotherms of N_2_ molecules at liquid nitrogen temperature (− 196 ℃). To characterize the hydrophilicity of the coaxial scaffolds, 5 μL liquid droplet was carefully dropped on the surface of the coaxial scaffold (*n* = 3), and the WCA was tested using the sessile-drop technique (DSA 100, Krüss, Germany) under room temperature. Besides, the chemical composition of the coaxial scaffolds was analyzed by energy-dispersive spectrum (EDS). In order to investigate the mechanical properties of the scaffolds, an electronic universal testing machine (Hua Long Inc., China) was used according to GB/T 228-2010 standard [23]. During the test, the scaffolds were all cut into rectangular shape with an average area of 30 × 15 mm^2^. For each test, the samples were stretched at a speed of 20 mm min^−1^ along the orientation of the nanofibers (*n* = 3). The swelling ratio of the scaffolds (PGA, MPGA, NMPGA) was evaluated in PBS solution using a gravimetric method (*n* = 3). After drying to constant weight in a vacuum oven, the scaffolds with a size of 4 × 4 cm^2^ were immersed in PBS solution at pH 7.4 and 37 °C shaking for 48 h. The swelling ratio of the as-prepared scaffolds (*Q*) was calculated according to Eq. (1):

1 $$Q = \frac{{W_{w} - W_{d} }}{{W_{d} }} \times 100\%$$

where *W*_*d*_ is the weight of dry scaffold, and *W*_*w*_ is the weight of wet scaffold which was weighed after the water adsorbing on the surface was removed with filter paper (*n* = 3).

Similarly, the degradation rate of the as-prepared scaffolds (*R*) was also calculated according to Eq. (2):

2 $$R = \frac{{W_{0} - W_{1} }}{{W_{0} }} \times 100\%$$

where *W*_0_ is the weight of the pristine scaffold, and *W*_1_ is the weight of the scaffold which was lyophilized and weighed after soaking in the PBS solution for 0, 3, 7, 49, and 70 d (*n* = 3). Correspondingly, the morphologies of the scaffolds were observed by FE-SEM.

### In vitro NO and Cu Ions Release from NMHK and NMPGA

Before NO and Cu ions release experiment, the NMPGA was numbered and cut into a square with an average area of 2 × 2 cm^2^, and the weight was recorded. Similarly, the NMHK was also numbered and weighted by a certain mass. After that, the as-prepared samples (NMHK and NMPGA) were immersed into 30 mL PBS solution (pH = 7.4) at 37 °C in a shaker with a rotating speed of 80 rpm for 2 w. At each defined time point, 4 mL of the release medium was taken out for detection, and an equal volume of the fresh PBS solution was added. Then, the ultraviolet spectrophotometer (UV-300, Thermo Spectronic, USA) was used to evaluate the amount of NO released from the medium with Griess reagent at 520 nm absorption peak (*n* = 3) [15, 25]. The concentrations of Cu ions released from the NMHK and NMPGA were determined by inductive coupled plasma atomic emission spectrometry (ICP-AES, Optima 7000 DV, Perkin-Elmer, USA) (*n* = 3).

### Cell Culture

Human umbilical vein endothelial cells (HUVECs) were cultured in endothelial culture medium (No. 1001, Sciencell) containing 5% fetal bovine serum (FBS) (No. 0025, Sciencell), 1% endothelial cell growth supplement (No. 1052, Sciencell) and 1% penicillin/streptomycin solution (No. 0503, Sciencell) in an incubator at 37 °C, 5% CO_2_.

### Biocompatibility Assessment of Scaffolds

The scaffolds were sectioned and stuck onto 8 mm cell slides (Solarbio, China) and were treated subsequently in 48-well cell plates (Corning, USA). The scaffolds were first washed out with a large amount of deionized water and then disinfected via immersing in the 75% (v/v) ethanol for 2 h. After that, the PBS solution was used twice, each for 5 min to clear the residual ethanol. Then, 0.5 mL HUVECs suspension was dropped onto the surface of the scaffold at a density of 0.5 × 10^4^ cells per well and cultured in an incubator at 37 °C, 5% CO_2_ for 1, 3, and 7 d. The medium was changed every other day. At the predetermined time, cell viability was assessed by CCK-8 (Beyotime, China) following the manufacturer’s instructions. The value of optical density (*n* = 3) was measured using a microplate spectrophotometer (Epoch™, BioTek, USA) at 450 nm absorbance.

### Cell Morphology on the Scaffolds

The aforementioned scaffolds cultured with HUVECs for 1, 3, and 7 d were fixed by 2.5% glutaraldehyde (Solarbio, China). For scanning electron microscope (SEM, Phenom ProX, Netherlands), the scaffolds were then dehydrated with graded ethanol and sputtered with gold for 80 s at a current of 5 mA. For confocal laser scanning microscopy (CLSM, Nikon C2, Japan), the scaffolds were stained with fluorescein isothiocyanate (FITC)-Phalloidin (Solarbio, China) for cytoskeletons and 4′,6-diamidino-2-phenylindole (DAPI, Solarbio, China) for nuclei according to the manufacturer’s instructions.

### Cell Tube Formation Assay

The basement membrane matrix (BD Matrigel™) was thawed at 4 °C overnight and placed 50 μL at the bottom of a precooled 96-well plate in each well, then incubated at 37 °C, 5% CO_2_ for 30 min. HUVECs treated by PGA, MPGA and NMPGA leachate in advance were seeded in each well (1.5 × 10^4^). After 2 h incubation, the tubular structure of HUVECs was captured by a light microscope (DP72, Olympus, Japan) at low magnification. Then, the nodes, total branching length, and circles were analyzed and quantified by Image J (NIH, USA) (*n* = 3). Red circles present the nodes; blue meshes present the circles. Purple lines and green lines together present the total branching length.

### Animal Experiment

The animal experiments were approved by the Animal Welfare and Ethics Group, Department of Laboratory Animal Science, Fudan University (No. 201904004Z). Ninety-six male Sprague–Dawley rats were randomly divided into four groups (Control, PGA, MPGA, NMPGA). The rats were 6 w old and 190 ~ 210 g in weight. All rats were anesthetized through intraperitoneal injection by chloral hydrate (300 mg kg^−1^). Under anesthesia, both the patellar tendons were exposed, then a 7 × 2 mm^2^ full-thickness window defect was created in the central third of the patellar tendon without bony defect [26]. Subsequently, a 7 × 2 mm^2^ scaffold (PGA, MPGA, NMPGA) was inserted. The fiber direction of the scaffold was consistent with the long axis of the patellar tendon. There is no scaffold in the Control group. After that, a 6-0 silk suture was used to sew up the patellar tendon defect and fix the scaffold at the same time. The wound was then irrigated with saline, and the skin was closed.

### Contrast-Enhancement Ultrasound Examination (CEUS) in vivo

At the predetermined time, the rats (*n* = 3) were anesthetized, and the hair around the knee was shaved. The CEUS examination was performed by two operators together (a sonographer with 10 y of experience and an animal operator to inject agent). CEUS imaging was acquired by an Aplio i900 (Canon Medical Systems, Japan) with an 18-MHz linear transducer. The transducer was placed on the skin softly so as not to compress the tissue. The plane with patella and tibia with the largest cross-sectional area of the patellar tendon were selected. The machine parameters were adjusted so that the mechanical index was 0.08, the frame rate was 10 fps, the gain was 60, and the dynamic range was 60 dB. After 0.2 mL SonoVue® (sulfur hexafluoride microbubble, a commercial ultrasound contrast agent) being injected from the tail vein, videos were recorded immediately for 90 s [27]. The digital imaging and communications in medicine (DICOM) data were analyzed by specific software (Time curve analysis V3.7, Canon, Japan) for PI and AUC. The region of interest in the video was a 9.0 × 2.5 mm^2^ ellipse (Fig. S2).

### Immunohistochemistry and Histopathological Analysis

At the predetermined time, patellar tendons were harvested and fixed in 4% paraformaldehyde immediately. Samples were gradually dehydrated, embedded in paraffin, sectioned to 4 μm in thickness, and stained with CD31, H&E, and PSR. For immunohistochemistry, sections were stained using a primary antibody specific for the CD31 (1:100, Abcam, UK), which was a marker of vascular endothelial cells. The CD31-positive staining area (*n* = 3) and the microvessel diameter (*n* = 10) were calculated. The PSR staining sections were observed from the same view under a circularly polarized microscope (DM2500P, Leica, Germany). For PSR staining, thick collagen fiber shows strong birefringence, which is yellow, and thin collagen fiber shows weak birefringence, which is green [28]. Both the yellow and green collagen fiber area proportion adjacent to the scaffolds were quantitatively analyzed under high magnification. To get rid of the influence of scaffold and interstitial space, the ratio of yellow collagen fibers proportion versus green collagen fibers proportion was also calculated (*n* = 3). The other two staining sections were observed under a light microscope (DP72, Olympus, Japan). All images were analyzed by Image J.

### Biomechanical Test

At the predetermined time, rats were sacrificed by overdose intraperitoneal injection. The bone-patellar tendon-bone complex was harvested. The patellar tendons were all trimmed to 4 mm width, and the cross-sectional areas of the patellar tendon were measured by Vernier calipers. The complex was tested for biomechanics using a universal mechanical instrument (Instron 5966, USA). The complex was fixed in the load frame with the fiber direction of the patellar tendon consistent with the long axis of the instrument. The ultimate load to failure was conducted at a speed of 2 mm min^−1^ and recorded using the software kit (Bluehill Universal, USA). The definition of failure was ruptured of the patellar tendon. The failure load (N) and tensile strength (MPa) were measured from the load-deformation curve (*n* = 5).

### Statistical Analysis

All quantitative results were expressed as mean ± SD. Two-way ANOVA with Tukey’s test and unpaired *t*-test were used to compare any significant difference between groups at different time points. GraphPad Prism Software v8.1 (San Diego, US) was used for the statistical analysis. *P* < 0.05 was considered statistically significant.

## Results and Discussion

### Characterization of NMHK and NMPGA

**Fig. 1:**
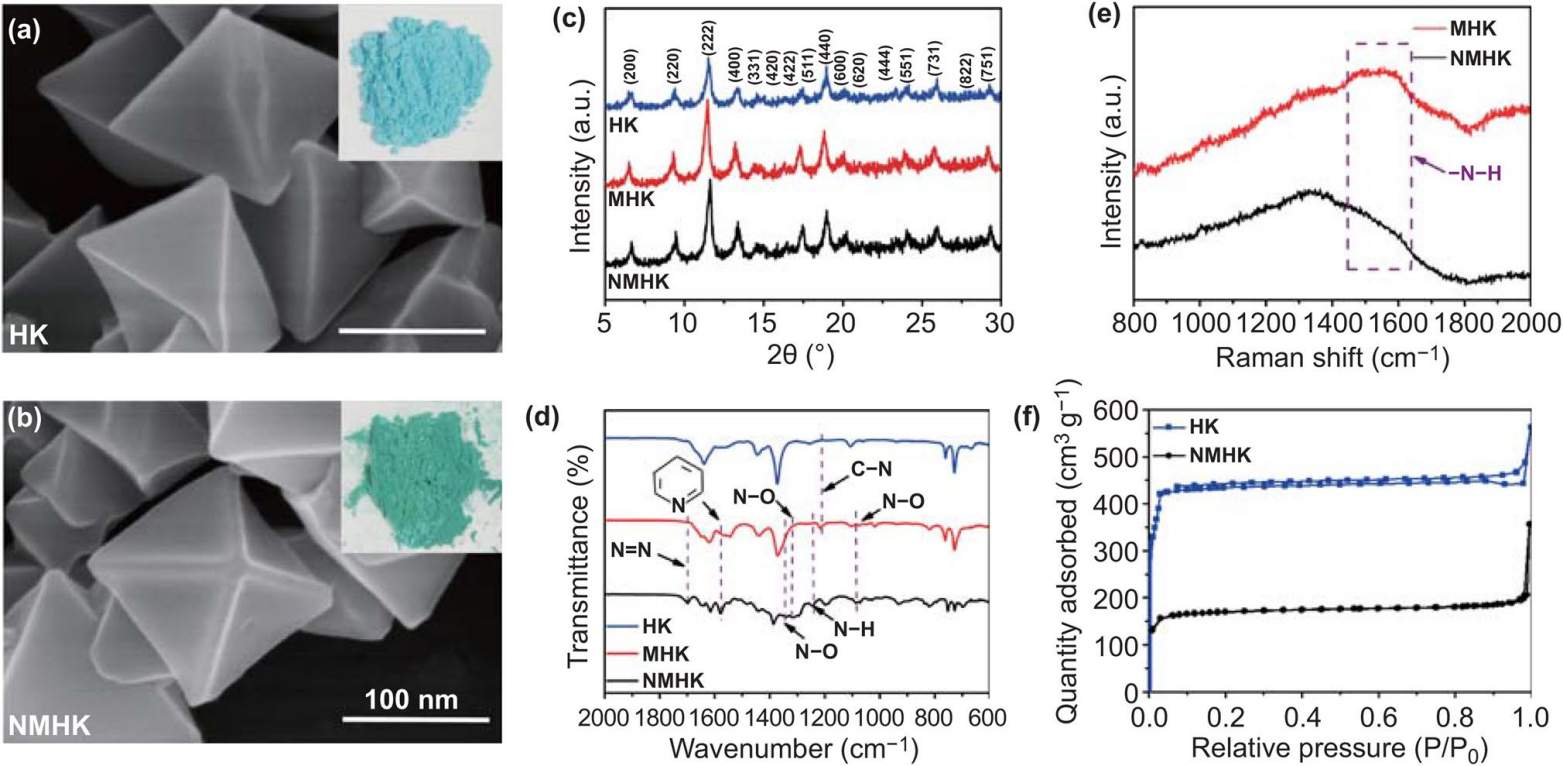
Characterization of the HK before and after modification. SEM images of HK **a** before and **b** after NO loading, the photographs inserted in each SEM showed the color of the HK before and after NO loading. **c** X-ray diffraction spectrum, **d** FTIR spectrum of the HK, MHK, and NMHK. **e** Raman spectrum of the MHK and NMHK. **f** Nitrogen isotherms of the HK and NMHK

The morphology and structural characterization of the HK and NMHK are shown in Fig. 1. As shown in the scanning electron microscope (SEM) images and photographs (inserted in each SEM image in Fig. 1a, b), it was clearly observed that the synthesized HK and NMHK exhibited quite uniform octahedral structures with sharp edges, and the quantitative analysis further exhibited that the average diameter of the nanoparticles was about 153.96 ± 12.53 nm and 165.79 ± 14.69 nm, respectively (Fig. S3). Moreover, even though the color of HK had changed from light blue to green after NO loading, the NMHK exhibited a stable octahedral morphology.

Additionally, the crystal phases of the as-prepared HK, 4-map modified HK (MHK), and NMHK were examined by X-ray diffraction (XRD), respectively. As depicted in Fig. 1c, the typical characteristic diffraction peaks appeared at 2*θ* of 6.71º, 9.52º, 11.70º, 13.46º, 17.51º, and 19.00º, which are indexed as the (200), (220), (222), (400), (511), and (440) planes of the typical HK structure [21, 29]. Furthermore, the positions of the diffraction peaks of the MHK and NMHK were the same as HK after 4-map modification and NO loading, and the crystal structure of NMHK had no significant change when compared with the HK (Fig. S4). These results indicated that the HK had been successfully synthesized, and the crystalline phases of HK had no significant change after the 4-map modification and NO-loading.

The Fourier-transform infrared spectrum (FTIR) in Fig. 1d further showed that, compared with the HK, there were several new peaks appeared at 1250 and 1542 cm^−1^ in the spectrogram of MHK, which are ascribed to the stretching vibration adsorption of the C-N bonds and the pyridyl group in the 4-map [22, 30]. Additionally, there were other new peaks, like 1129, 1250, 1290,1350, and 1600 cm^−1^ appeared in the NMHK, which were assigned to the stretching vibration of the N–O, N–H, N–O, N–O, and N = N bonds, respectively [31, 32]. Besides, as shown in Figs. 1e and S5, the Raman spectrum showed that the peak at 1400 ~ 1600 cm^−1^ in MHK, which corresponds to the secondary amino groups (-N–H), was disappeared after NO-loading, which may be due to the combination of -N–H groups with NO [31]. All these results demonstrated that the HK had been successfully modified by 4-map, and the NO had been successfully loaded in the MHK.

As shown in Figs. 1f and S6, both the HK and NMHK exhibited classical type-IV isotherm curves, indicating the existence of the uniform microporous channel structures inside the HK and NMHK [33]. Moreover, it could also be noticed that the N_2_ adsorption–desorption analysis in Table S1 further showed that the specific surface area, the average pore diameters, and the total pore volume of HK were 1194.77 m^2^ g^−1^, 21.22 Å, and 0.49 mL g^−1^, respectively. However, the specific surface area, average pore diameter, and total pore volume of NMHK were remarkably decreased, and the corresponding results were 609.48 m^2^ g^−1^, 20.55 Å, and 0.34 mL g^−1^, respectively, which were attributed to the 4-map modification and NO-loading. All these aforementioned characteristic results indicated that not only 4-map had successfully been modified onto the HK but also NO had successfully been loaded.

**Fig. 2:**
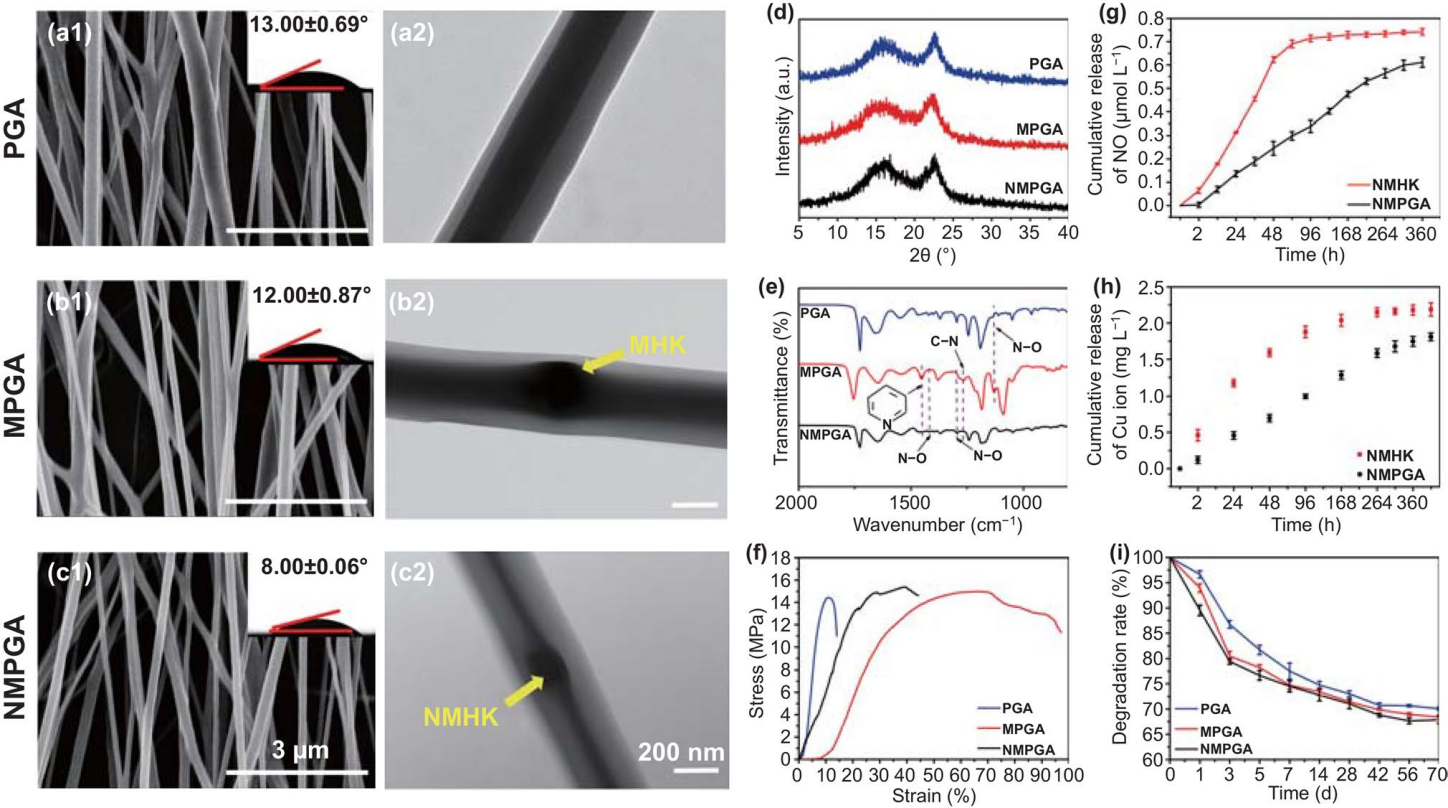
Characterization of the aligned coaxial scaffolds. SEM images of the **a1** PGA, **b1** MPGA, and **c1** NMPGA, and the photographs inserted in each SEM image showed the corresponding WCA. TEM images of the **a2** PGA, **b2** MPGA, and **c2** NMPGA. **d** X-ray diffraction spectrum, **e** FTIR spectrum, and **f** stress–strain curve of the PGA, MPGA, and NMPGA. Cumulative release of **g** NO and **h** Cu ions from the NMHK and NMPGA in PBS solution. **i** Degradation rate of the PGA, MPGA and NMPGA

The morphology and structural characterization of the aligned coaxial scaffolds were characterized by a number of techniques. The morphologies of the PCL/Gel aligned coaxial scaffolds (PGA), MOFs encapsulated in PCL/Gel aligned coaxial scaffolds (MPGA), and NMPGA under SEM are shown in Fig. 2a1–c1. All the scaffolds showed well-organized topological structures with nanofibers tending to arrange in a parallel manner, which could be recognized as aligned [33]. Moreover, the average diameter of the nanofiber was about 365.26 ± 31.98 nm for PGA, 379.27 ± 39.05 nm for MPGA, and 390.78 ± 28.15 nm for NMPGA (Fig. S7), which demonstrated that the nanofibers exhibited a uniform diameter distribution and the incorporation of MHK and NMHK had little effect on the diameter of the nanofibers. Besides, the water contact angle (WCA) showed the hydrophilicity of the scaffolds. The WCA of the PGA, MPGA, and NMPGA was 13.00 ± 0.69°, 12.00 ± 0.87°, and 8.00 ± 0.06°, respectively, revealing that the scaffolds were quite hydrophilic, which can further reveal the good biocompatibility of our scaffolds [34]. Furthermore, the swelling ratio of the as-prepared scaffolds (PGA, MPGA, NMPGA) after being immersed in phosphate-buffered saline (PBS) solution for 48 h was investigated in response to physiological conditions (Fig. S8). For each sample, about three times burst swelling of dry weight was observed. The swelling ratio could reflect the hydration process of the coaxial scaffolds. As one of the important factors, the surface wettability would have a large effect on the swelling ratio under the same raw material and swelling medium [35]. In this study, the WCA showed the hydrophilicity of the scaffolds, which resulted in a higher hydration degree and a higher swelling ratio.

To evaluate whether MHK and NMHK had been well wrapped inside the aligned coaxial nanofibers or not, the transmission electron microscopy (TEM) was initially used to exam the structure of nanofibers. As displayed in Fig. 2a2–c2, the nanofibers in all the as-prepared scaffolds (PGA, MPGA, NMPGA) exhibited core–shell structures with no nanoparticle in the PGA and some nanoparticles in the MPGA and NMPGA. Additionally, XRD was used to detect the crystalline phases of the as-prepared scaffolds. As shown in Fig. 2d, due to the shielding effect of the PCL and Gel, the XRD spectrum of PGA, MPGA, and NMPGA presented two amorphous phases state [36]. The FTIR shown in Fig. 2e revealed that, excepting for the characteristic peaks of PCL and Gel, there were new peaks, which were assigned to the stretching vibration of the N–O, C-N, N–O, N–O, and pyridyl group bonds, found in the MPGA and NMPGA samples. Furthermore, the element mapping and energy dispersive spectrum (EDS) shown in Fig. S9 demonstrated that C, N, and O signals were evenly distributed in PGA, MPGA, and NMPGA, but the Cu signal could only be detected in the MPGA and NMPGA. Therefore, it could be concluded that the MHK and NMHK nanoparticles had been successfully embedded into the nanofibers.

As tendons are load-bearing structures, mechanical performance is one of the necessary requirements for the tendon scaffolds. As shown in Fig. 2f, the stress–strain curve demonstrated that all the PGA, MPGA, and NMPGA possessed high stress with low strain, which was caused by the orderly structure. Moreover, the mechanical properties of PGA, MPGA and NMPGA are shown in Fig. S10. The results showed that there was no significant difference for the max load, tensile strength, and Young’s modulus among the three groups. The mechanical results indicated that PCL/Gel is the primary mechanical provider for the obtained PGA, MPGA and NMPGA. Notably, the testing value in all three groups met both the United States and British Pharmacopeia tensile strength requirements for suture material [37]. To sum up, these three scaffolds could be considered as acceptable scaffolds in the repairing of tissue injury.

The cumulative drug release experiments were conducted in vitro to examine the efficacy of NMHK and NMPGA as vehicles. The concentration of NO released from the NMHK and NMPGA is shown in Fig. 2g. Regarding the physiological concentration of NO in the body, compared with the concentration of NO released by NMHK in the initial 48 h (about 0.6 μM), which could result in cell cycle arrest [38, 39], the concentration of NO in the NMPGA decreased to approximately 0.25 μM in this study, which could benefit for vasodilatory and angiogenic effects [40]. Moreover, the release of NO in NMPGA could reach 15 d with a slow release rate of nearly 1.67 nM h^−1^, and the NO concentration at 15 d was about 0.60 μM. All the above results indicated that the NMPGA could release NO with a controllable and reasonable concentration, which could improve the efficacy of NO as a tendon therapeutic agent during the tendon repairing process. As shown in Fig. 2h, the concentration of Cu ions released from the NMHK and NMPGA was also analyzed. Similar to the release of NO, the release of Cu ions was also slower in NMPGA when compared with that in NMHK, which would accelerate the healing process [41–43]. Thus, the release results demonstrated that NMPGA could provide a suitable microenvironment for tissue regeneration by controlling the release rate of NO and Cu ions.

As a kind of tissue repair material, the implantation needs to have an appropriate degradation rate. Herein, the degradation rate of the aligned coaxial scaffolds has been investigated. As depicted in Fig. 2i, it was observed that all the as-prepared scaffolds (PGA, MPGA, NMPGA) were degraded to less than 80% within 7 d, which might be due to the degradation of Gel. After 8 w, the degradation ratio remained motionless, which indicated that the Gel contained in the scaffolds had almost been degraded. In order to further evaluate the degradation performance of the scaffolds, the morphology of the degraded NMPGA is shown in Fig. S11. Compared with 0 d, the nanofiber morphologies in 3, 7, 49, and 70 d began changing from well-defined to swollen, which is due to the degradation of Gel. Additionally, after the degradation of Gel on the nanofibers, PCL became clearer, which would suffer from a more prolonged degradation according to the previous study [44]. The degradation results demonstrated that the as-prepared scaffolds could support the tendon regeneration as long as 70 d.

### Proliferation, Morphology, and Tubular Formation of HUVECs Treated with NMPGA in vitro

**Fig. 3:**
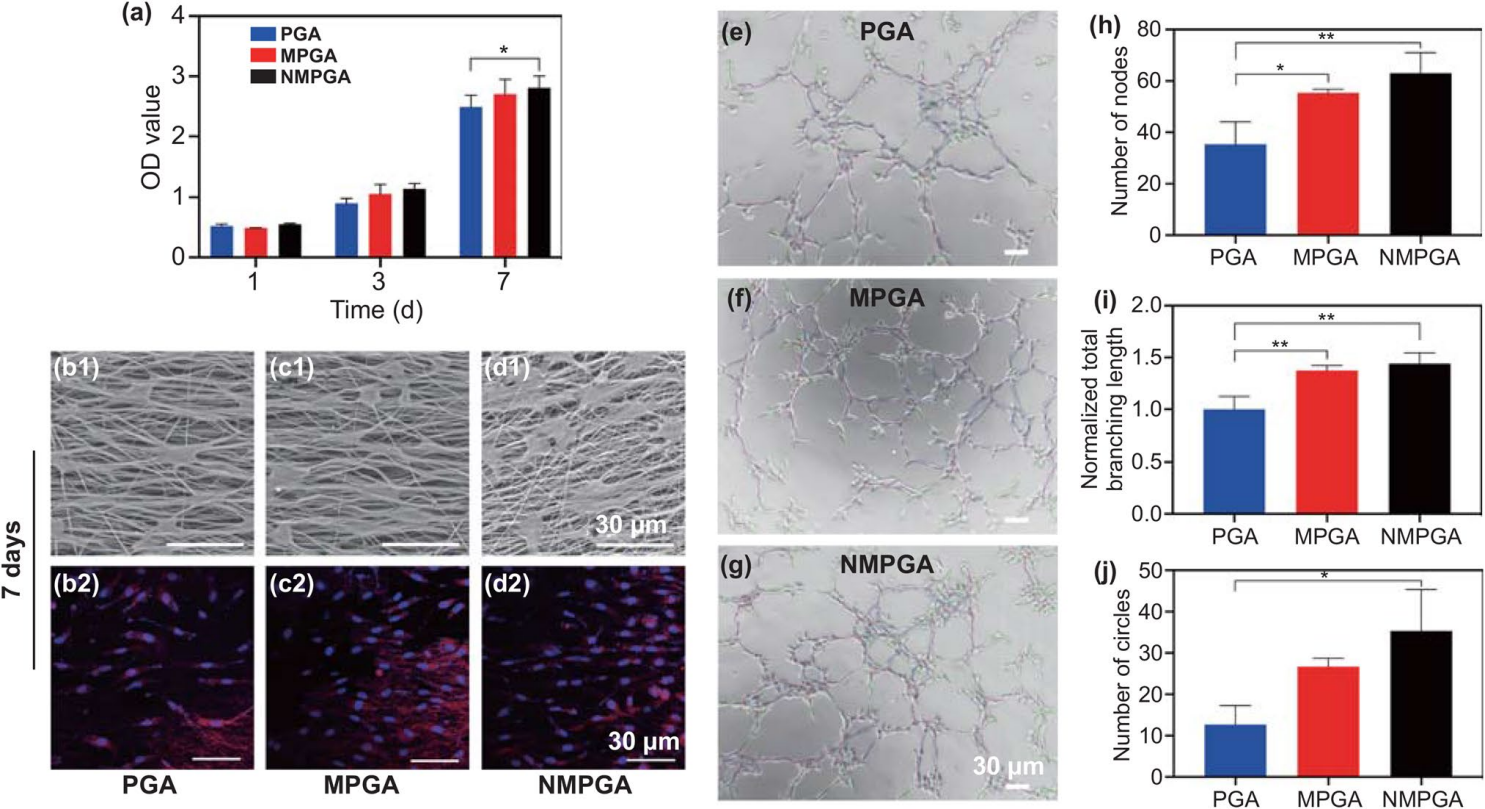
Proliferation, morphology, and tubular formation of HUVECs in vitro. **a** OD value of HUVECs at 450 nm of the PGA, MPGA, and NMPGA group after being cultured for 1, 3, and 7 d. Representative **b1, c1, d1** SEM images and **b2, c2, d2** CLSM images of HUVECs cultured on the PGA, MPGA, and NMPGA for 7 d. FITC-Phalloidin for cytoskeletons (red) and DAPI for nuclei (blue). **e–g** Representative bright-field images of HUVECs at 2 h in tubular formation assay in the PGA, MPGA, and NMPGA group. The quantitative analysis of the **h** number of nodes, **i** normalized total branching length, and **j** number of circles (Scale bar = 30 μm, ^*^P < 0.05). (Color figure online)

Firstly, cell counting kit-8 (CCK-8) was employed to examine the growth of human umbilical vein endothelial cells (HUVECs) cultured with each scaffold (PGA, MPGA, NMPGA). As shown in Fig. 3a, all O.D values of HUVECs cultured with three scaffolds increased as the culture time increasing, suggesting all the tested scaffolds had good biocompatibility. Furthermore, it was observed that the O.D value of the NMPGA group was significantly higher than that of the PGA group at 7 d (PGA vs. MPGA, *P* = 0.1808; PGA vs. NMPGA, *P* = 0.0322; MPGA vs. NMPGA, *P* = 0.6377), which might be mainly attributed to the introduction of Cu ion and NO [45, 46].

Secondly, the morphology of HUVECs on the three aligned coaxial scaffolds was observed by SEM and confocal laser scanning microscope (CLSM), respectively. The HUVECs spread on the surface of all three scaffolds, and there is no obvious difference in the morphology of HUVECs on the three scaffolds at 1, 3 (Fig. S12), and 7 d (Fig. 3b1, c1, d1). Then, the CLSM images showed that the cytoskeleton of most HUVECs stretched along the direction of fibers in all groups (Fig. 3b2, c2, d2). These results confirmed that all three scaffolds with orderly structures could guide the growth direction of HUVECs, which were in agreement with our previous observation [23].

**Fig. 4:**
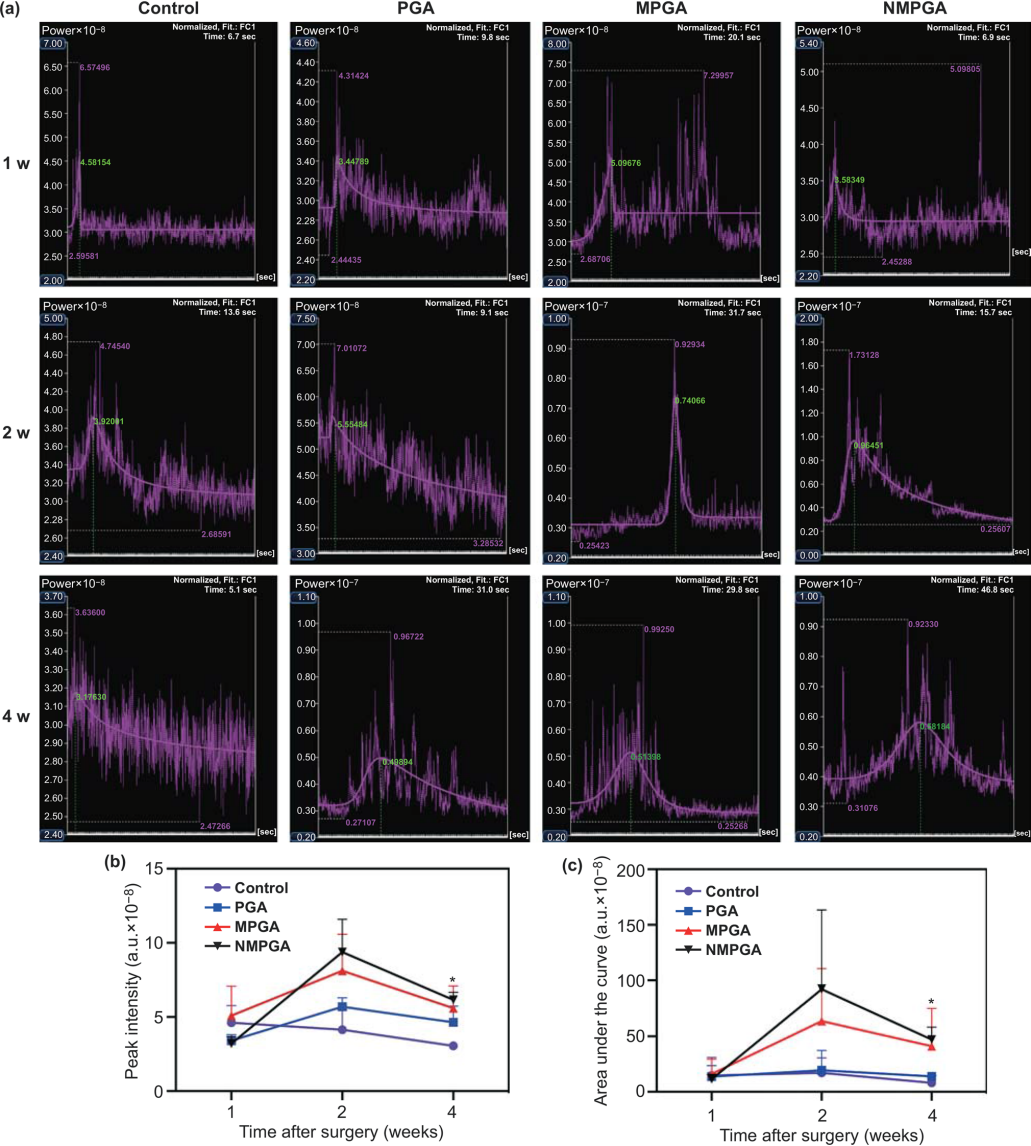
**a** Fitting time-intensity curves of the four groups (Control, PGA, MPGA, NMPGA) at 1, 2, and 4 w post-surgery. The quantitative analysis of the fitting time-intensity curves for **b** peak intensity and **c** area under the curve (*NMPGA vs. Control, *P* < 0.05)

Thirdly, a tubular formation assay was conducted in order to evaluate the in vitro angiogenic properties of PGA, MPGA and NMPGA. As observed in Fig. 3e–g, HUVECs in all three groups could form capillary-like network structures, while the NMPGA group demonstrated the most number of nodes (PGA vs. MPGA, *P* = 0.0279; PGA vs. NMPGA, *P* = 0.0064; MPGA vs. NMPGA, *P* = 0.4161) and the longest normalized total branching length (PGA vs. MPGA, *P* = 0.0077; PGA vs. NMPGA, *P* = 0.0034; MPGA vs. NMPGA, *P* = 0.6930) among three groups (Fig. 4h, i). Furthermore, in terms of the circle (Fig. 4j), a more complex morphological structure of angiogenesis in tubular formation assay [47], NMPGA group also showed the most number of circles comparing with PGA and MPGA (PGA vs. MPGA, *P* = 0.0848; PGA vs. NMPGA, *P* = 0.0123; MPGA vs. NMPGA, *P* = 0.3019). The tubular formation assay results demonstrated that the angiogenic properties were further improved in NMPGA group loaded with NO, which would benefit in repairing the injured tendon.

### Blood Microcirculation Evaluation in vivo

As a real time, dynamic detection method for blood microcirculation, contrast-enhancement ultrasound (CEUS) was applied to evaluate the blood perfusion of the repaired patellar tendon in vivo. The fitting time-intensity curves (TIC) detected by CEUS depicted the change of blood perfusion signal intensity with time, which demonstrated the blood microcirculation near the injury site in the detection period. At 1 w post-surgery, TIC of the PGA, MPGA and NMPGA group (Fig. 4a) was as poorly defined as the Control group. However, the TIC of both the MPGA group and NMPGA group appeared bell-shaped as early as 2 w post-surgery, while it was finally formed in the PGA group at 4 w post-surgery. This result revealed that both the MPGA group and NMPGA group could recover the regular wash-in and wash-out pattern of blood perfusion earlier than those in the Control group and PGA group [48], which could be ascribed to the angiogenic effect of Cu ions [43]. Besides, the quantitative analysis of peak intensity (PI) and area under the curve (AUC) based on the TIC is displayed in Fig. 4b, c. The PI and AUC represents the transient and a period of blood perfusion volume, respectively. Both PI and AUC in the MPGA group and NMPGA group demonstrated higher mean values than the Control group and PGA group since 2 w. Furthermore, both the PI and AUC in the NMPGA group were the highest among four groups and were significantly higher than the Control group at 4 w, which suggested that the addition of NO could further enhance the blood perfusion near the injury site [49]. In a word, compared with other groups, the recovery period of injured tissue in the NMPGA group was accelerated by early recovering the blood supply during tendon repair.

### Immunohistochemistry Evaluation of Angiogenesis ex vivo

**Fig. 5:**
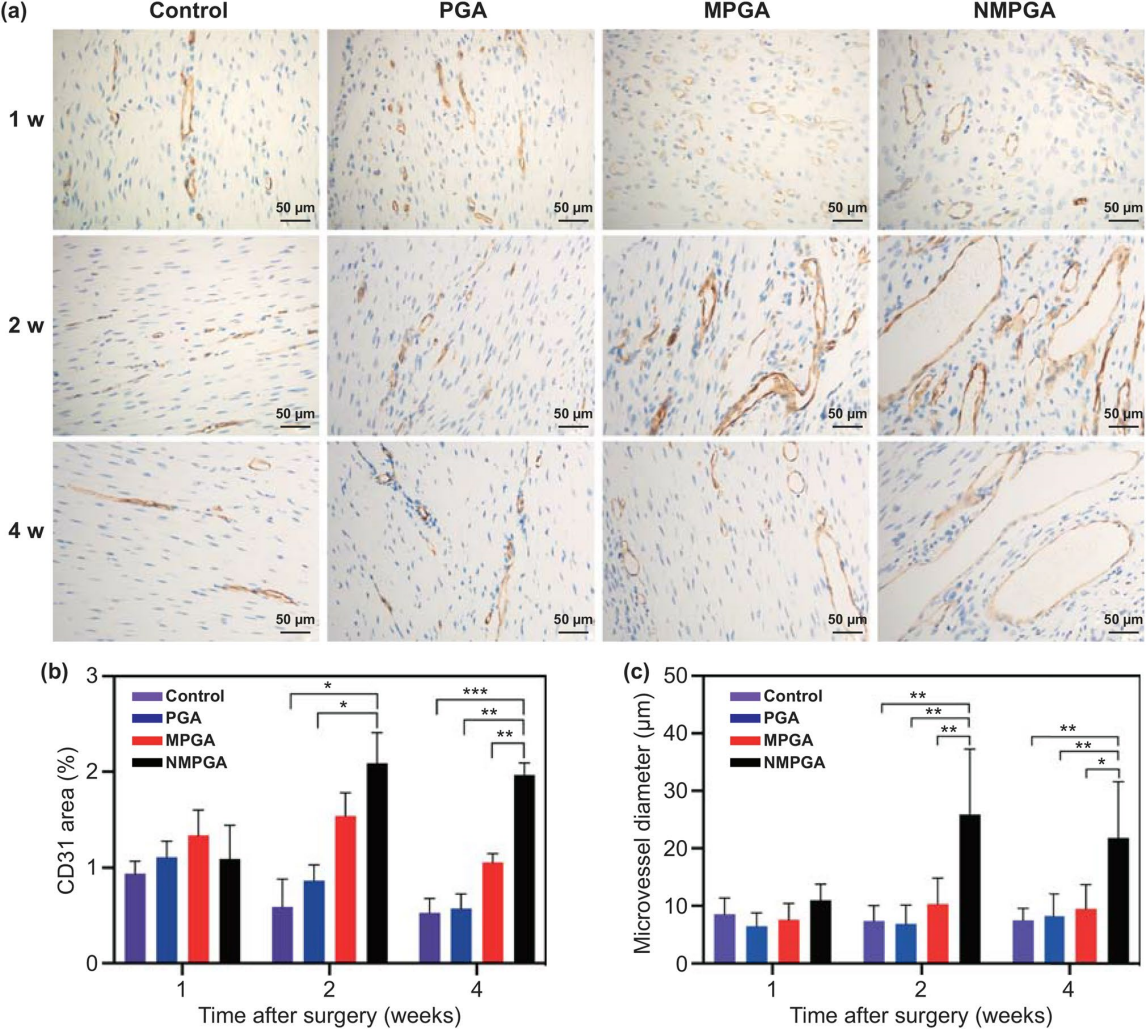
**a** Representative CD31 staining images in the healing patellar tendon of the Control, PGA, MPGA, and NMPGA group at 1, 2, and 4 w post-surgery. Quantitative analysis of **b** CD31-positive staining area and **c** microvessel diameter at 1, 2, and 4 w post-surgery (Scale bar = 50 μm, *P < 0.05, **P < 0.01, ***P < 0.005)

Additionally, the angiogenic property of the four groups (Control, PGA, MPGA, NMPGA) was also evaluated ex vivo by immunohistochemistry with vascular endothelial cell marker CD31 post-surgery. As shown in Fig. 5a, although no obvious angiogenic signs appeared in four groups in previous CEUS evaluation at 1 w, CD31-positive cells could still be detected in all tested sections as earlier as 1 w. With the increase in time, more and more CD31-positive cells could be observed in all groups, and some CD31-positive microvessels could be clearly visualized in the MPGA group and NMPGA group since 2 w. Furthermore, the CD31 positive-staining area proportion and microvessel diameter were quantitatively analyzed. As shown in Fig. 5b, c, both the CD31 positive-staining area proportion and microvessel diameter in the NMPGA group were significantly larger than those in other groups at 2 and 4 w, which may be due to the angiogenic and vasodilatory effects of NO [50]. The immunohistochemistry analysis results further confirmed that NMPGA could promote local blood supply by inducing the mature of neovascularization, which was consistent with our previous CEUS results.

### Histopathological Analysis and Biomechanical Evaluation of the Regenerated Tendon

**Fig. 6:**
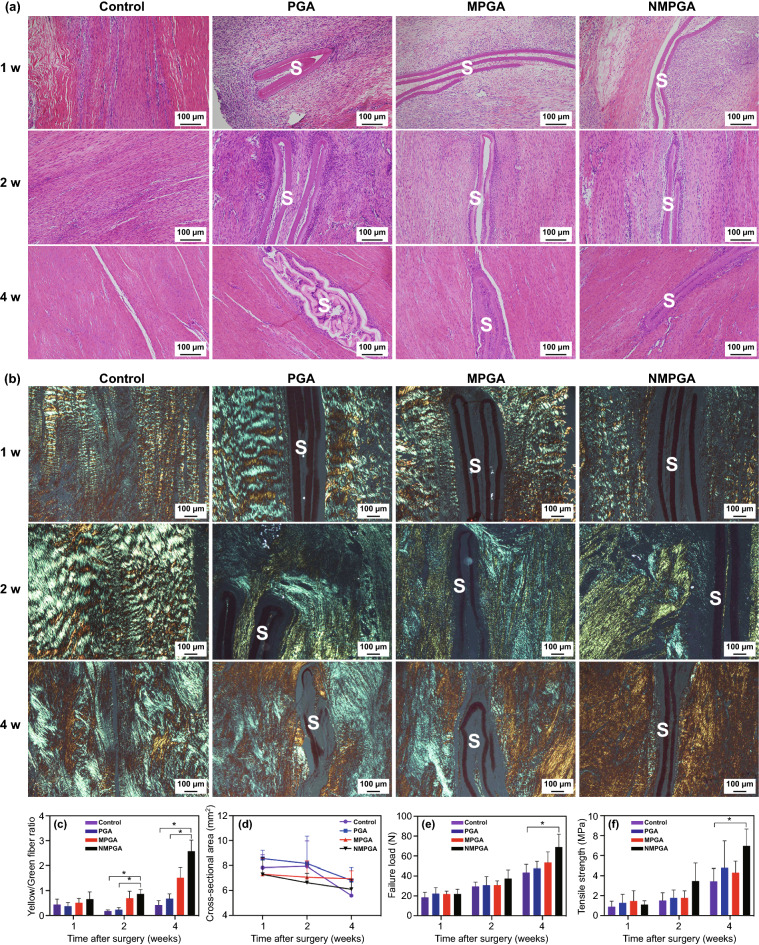
Histopathological analysis and biomechanical properties of the regenerated tendon in the Control, PGA, MPGA, and NMPGA group at 1, 2, and 4 w post-surgery. Representative **a** H&E staining images and **b** PSR staining images. **c** Quantitative analysis of the PSR staining for the ratio of yellow fibers versus green fibers. Quantitative analysis of **d** cross-sectional area, **e** failure load, and **f** tensile strength of the regenerated tendon (S = Scaffold, Scale bar = 100 μm, *P < 0.05). (Color figure online)

Finally, the healing quality of the regenerated tendon was assessed. As shown in Fig. 6a, hematoxylin–eosin (H&E) staining showed that the scaffolds remained existed in PGA, MPGA and NMPGA groups for 4 w. Notably, the foreign body reaction around the scaffolds in the NMPGA group was milder than that in the PGA group at 1 and 2 w post-surgery, which could be attributed to the anti-inflammatory mechanism of NO [51]. Besides, the picrosirius red (PSR) staining was quantitatively analyzed for appraising collagen networks in adjacent tissues to the scaffolds. As shown in Fig. 6b, yellow collagen fibers referred to collagen type I and green collagen fibers referred to collagen type III [52]. The area proportion of collagen type I increased continuously in the NMPGA group (Fig. S13), which indicated the increasing maturity of the collagen fibers [53–56]. Furthermore, as exhibited in Fig. 6c, the mean yellow/green fiber ratio in the NMPGA group was the highest among the four groups after surgery. It showed a significant increase compared with the Control group since 2 w, which further indicated the increasing maturity of the regenerated collagen induced by NMPGA. Moreover, as shown in Fig. 6d, the cross-sectional area decreased in all groups at 4 w post-surgery compared with 1 w, which demonstrated that the remodeling of collagen fibers enhanced the fusion between regenerated tissue and adjacent host tissue [57]. Meanwhile, the collagen fibers of the NMPGA group could fuse faster than the PGA group and MPGA group during tendon repair, which suggested that NMPGA could accelerate the healing process of injured tendons.

Besides, as shown in Fig. 6e, f, the failure load and tensile strength in all groups displayed continuous increase since 1 w post-surgery, and both failure load and tensile strength in NMPGA group were the highest among four groups since 2 w, which could correlate with earlier maturity and fusion of collagen fibers [58, 59]. Thus, those results demonstrated that the regenerated tendon in the NMPGA group could recover in a shorter healing period with better biomechanical properties.

## Conclusions

In conclusion, a NO therapeutic nanosystem for tendon regeneration was successfully designed and constructed by combining the high NO payload capability of MOF materials with the highly aligned coaxial structure of PCL/Gel scaffold. Notably, our prepared NMPGA could release NO stably and slowly at nearly 1.67 nM h^−1^ as long as 15 d, without a burst release in the initial 48 h in vitro, and had a degradation period as long as 70 d, which could provide a suitable biological and mechanical microenvironment for repairing damaged tendon tissue. Furthermore, the NMPGA demonstrated excellent angiogenic and vasodilatory effects by promoting the tubular formation of HUVECs and rapidly increasing the blood perfusion near the rabbit patellar tendon injury site as early as 2 w post-surgery. Besides, the regenerated tendon after implanting NMPGA had maturer collagen fibers and better biomechanical properties in comparison with that in other groups. Overall, our study not only provides a promising NO-loaded scaffold candidate for tendon regeneration but also paves a novel strategy for developing a MOFs-based gas therapeutic system.

## Electronic supplementary material

Below is the link to the electronic supplementary material.

Supplementary file1 (PDF 1175 kb)
