# Supplementary material for: MOFs-Based Nitric Oxide Therapy for Tendon Regeneration
Source: Nanomicro Lett. 2020 Nov 11;13:23. doi: 10.1007/s40820-020-00542-x (PMC8187533; doi:10.1007/s40820-020-00542-x)
Supplement: Supplementary file 1 — Supplementary file1 (PDF 1175 kb) [file 40820_2020_542_MOESM1_ESM.pdf]

Supporting Information for

MOFs-Based Nitric Oxide Therapy for Tendon Regeneration

Jun Chen[1, #], Dandan Sheng[1, #], Ting Ying[2, #], Haojun Zhao[3], Jian Zhang[1], Yunxia Li[1], He Xu[2, *], Shiyi Chen[1, *]

[1]Department of Sports Medicine, Huashan Hospital, Fudan University, Shanghai 200040, People's Republic of China

[2]College of Chemistry and Materials Science, Shanghai Normal University, Shanghai 200234, People's Republic of China

[3]Department of Ultrasound, Jing'an District Center Hospital, Fudan University, Shanghai 200040, People's Republic of China

[#]Jun Chen, Dandan Sheng and Ting Ying contributed equally to this work

*Corresponding authors. E-mail: cshiyi@163.com (Shiyi Chen), xuhe@shnu.edu.cn (He Xu)

Supplementary Figures and Table

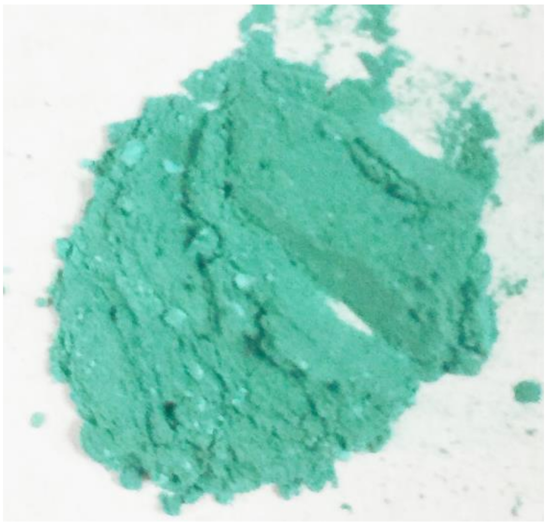

Fig. S1 Digital photo of the solid products MHK

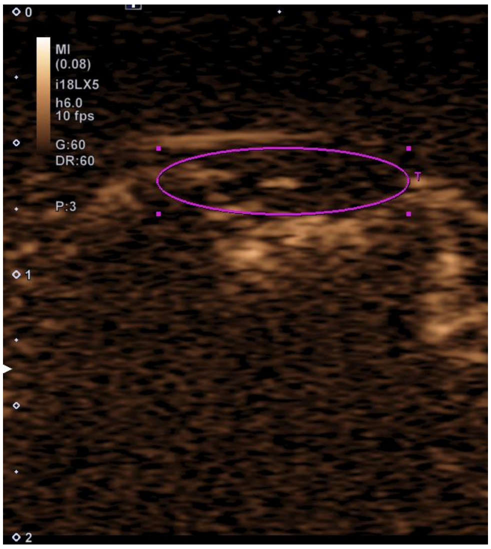

Fig. S2 CEUS imaging in the video. The purple circle presents the region of interest

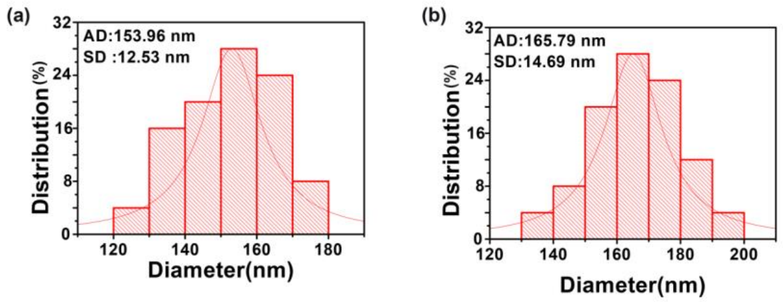

Fig. S3 Statistical analysis of the size distribution of (**a**) HK and (**b**) NMHK

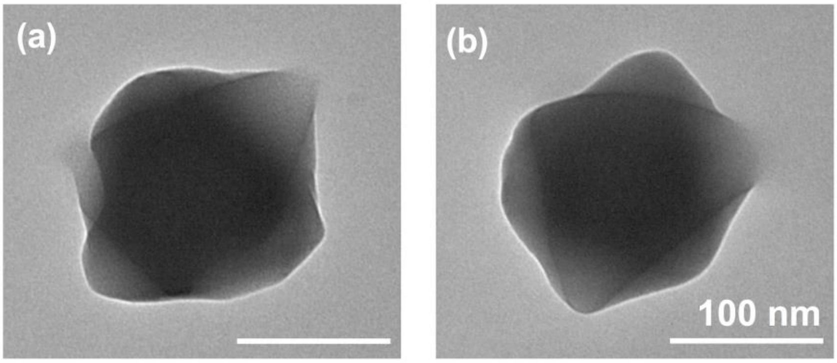

Fig. S4 TEM images of **a** HK and **b** NMHK (Scale bar = 100 nm)

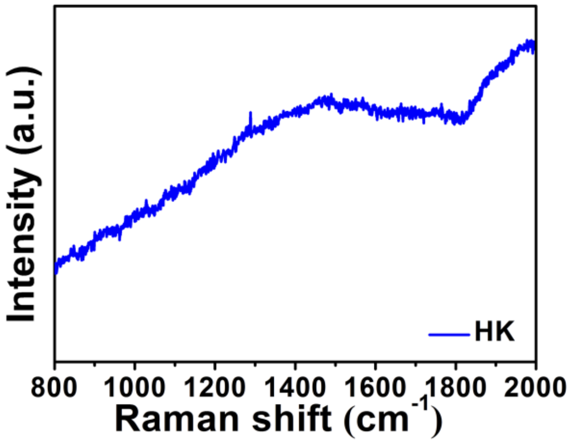

Fig. S5 Raman spectrum of the HK

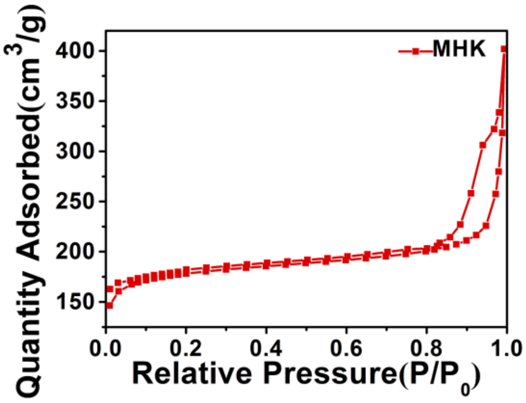

Fig. S6 Nitrogen isotherms of the MHK

Table S1 Structure parameters of HK and NMHK

	S_{BET} (m^2 g^{-1})	D_p (Å)	V_p (cm^3 g^{-1})
HK	1194.77	21.22	0.49
NMHK	609.48	20.55	0.34

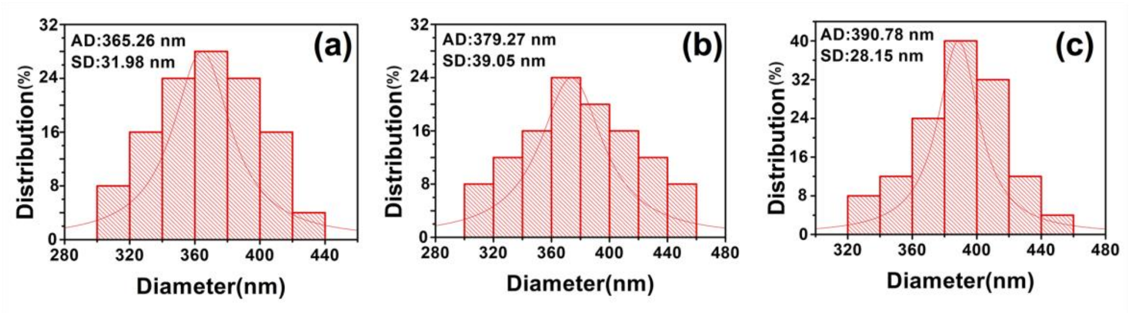

Fig. S7 Statistical analysis of the fiber diameter of the **a** PGA, **b** MPGA, and **c** NMPGA

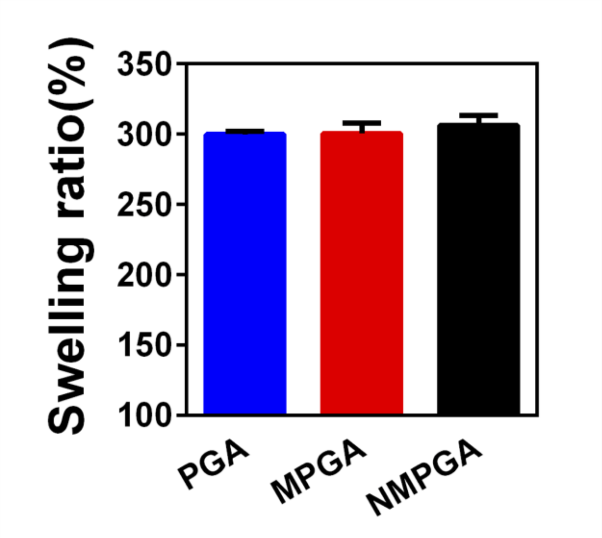

Fig. S8 Swelling ratio of the PGA, MPGA and NMPGA

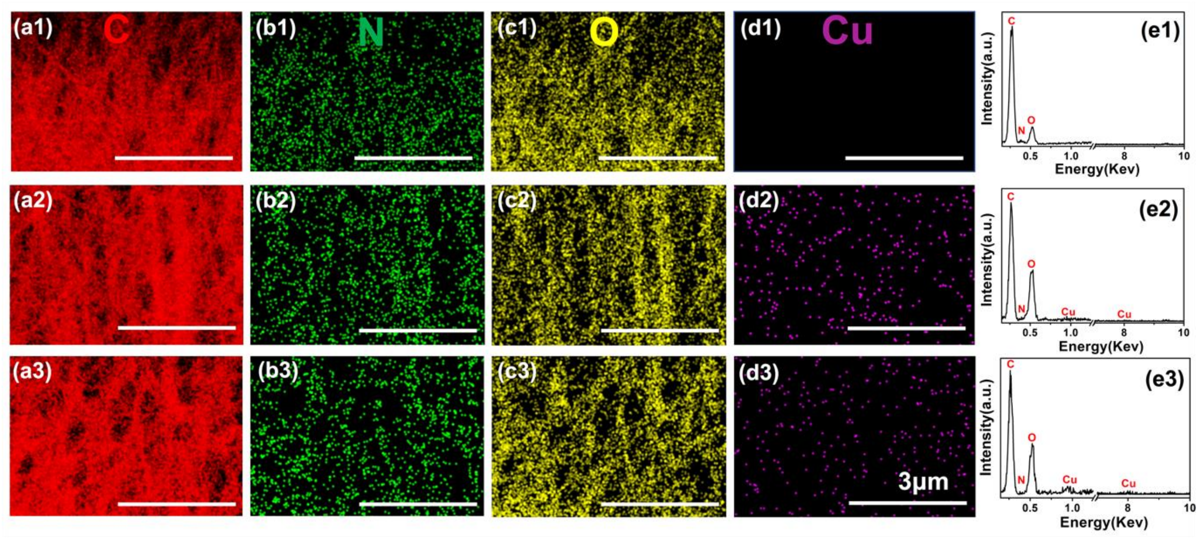

Fig. S9 Element mapping analysis showed C, N, O, Cu element distributed in the scaffolds: **a1-d1** PGA, **a2-d2** MPGA and **a3-d3** NMPGA. The intensity of element contents in the corresponding sample were shown in graph **e1**, **e2,** and **e3** (Scale bar = 3 μm)

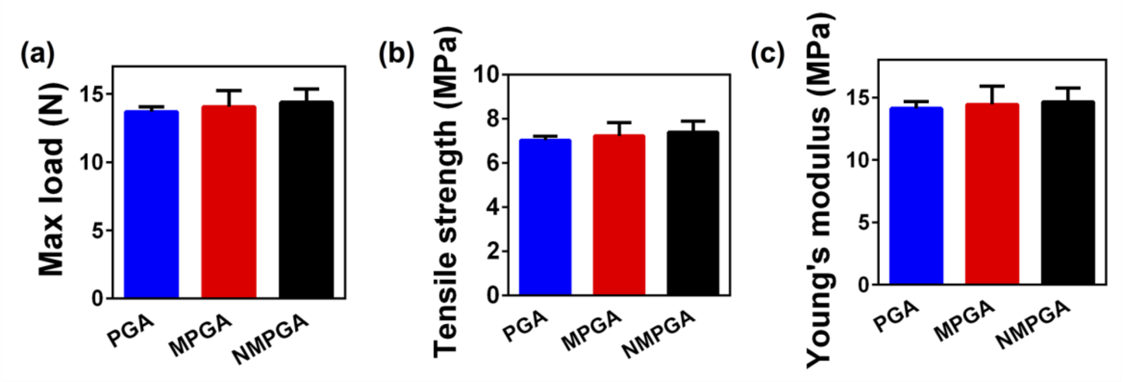

Fig. S10 **a** Max load, **b** tensile strength, and **c** Young's modulus of the PGA, MPGA, and NMPGA

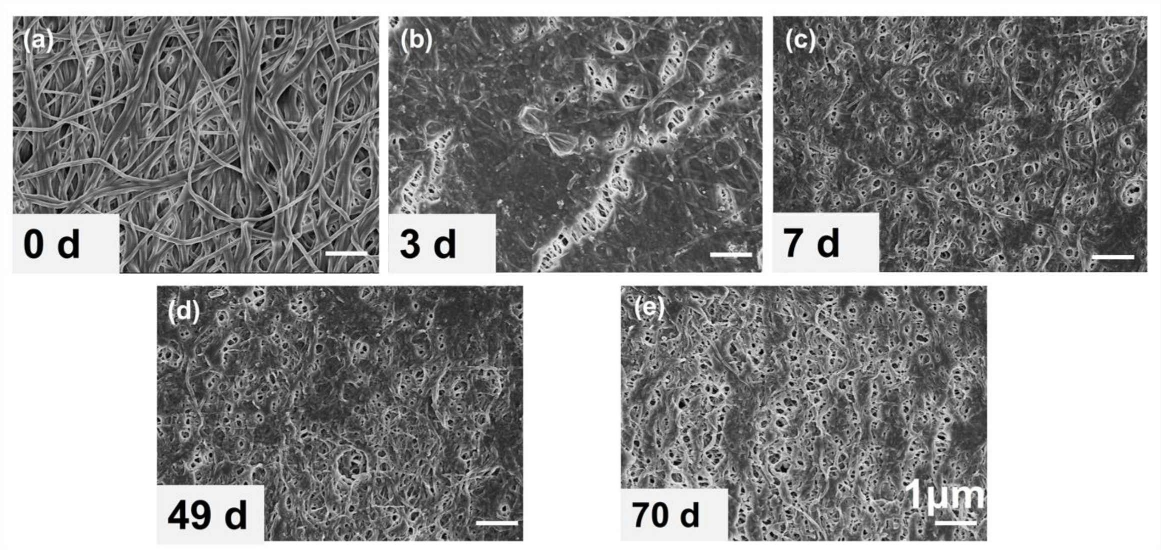

Fig. S11 FE-SEM images of the morphology of the NMPGA after soaking in PBS solution for 0, 3, 7, 49, and 70 d (Scale bar = 1 μm)

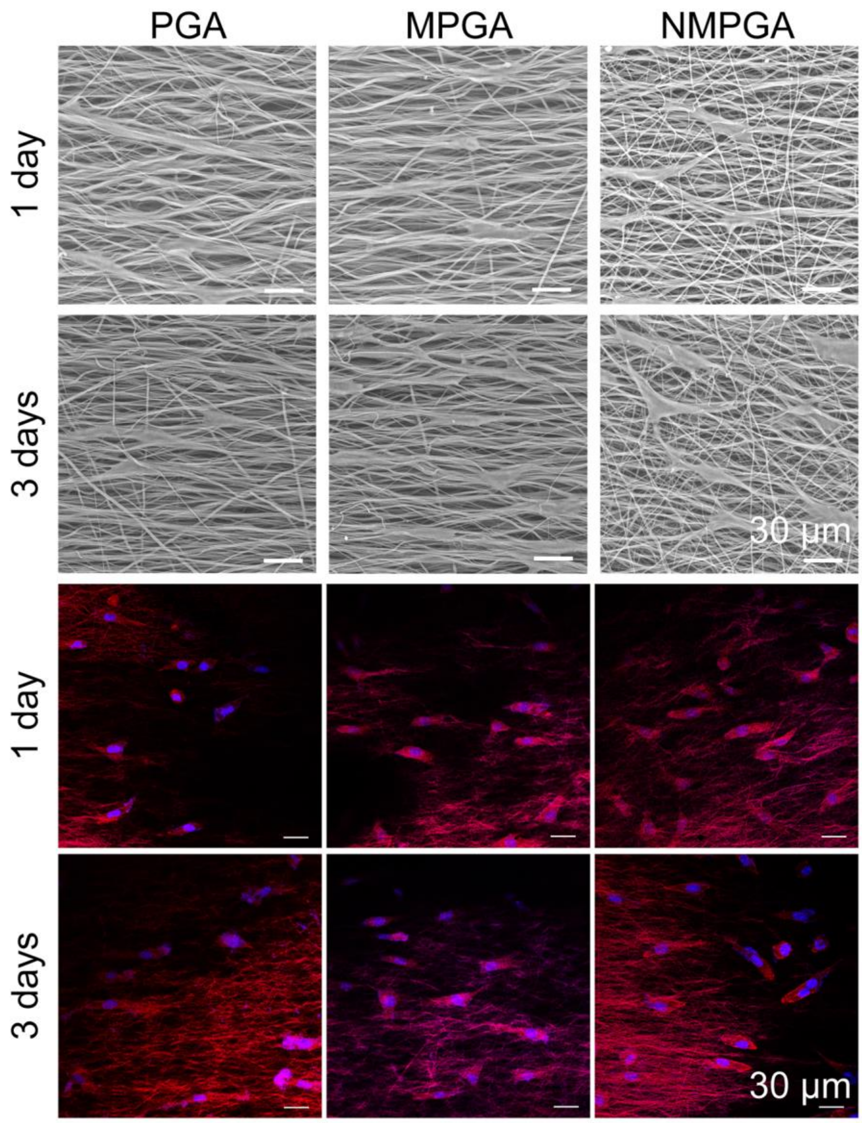

Fig. S12 SEM and CLSM images of HUVECs cultured on the PGA, MPGA, NMPGA for 1 and 3 d. FITC-Phalloidin for cytoskeletons (red) and DAPI for nuclei (blue) (Scale bar = 30 μm)

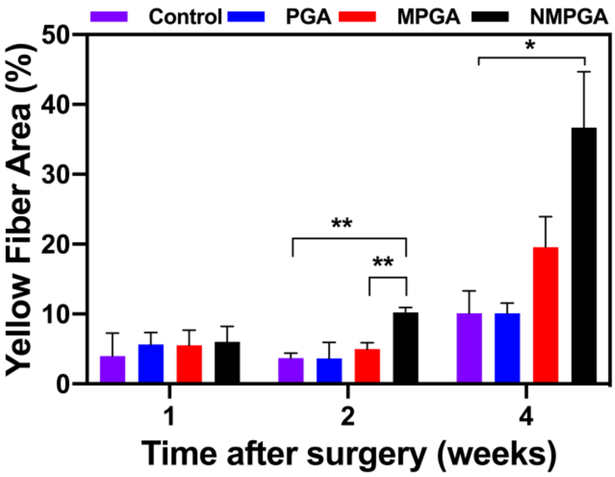

Fig. S13 Yellow collagen fiber area proportion at 1, 2, and 4 w post-surgery (*P < 0.05, **P < 0.01)
